# Kyste epidermoïde de la grande citerne et du quatrieme ventricule

**Published:** 2012-09-28

**Authors:** Noureddine Oulali, Faycal Moufid, Mohamed Rachid Ghailan, Brahim Hosni

**Affiliations:** 1 Department of Neurosurgery, Medical School, University Mohammed First, Oujda Morocco; 2 Department of Otolaryngology, Medical School, University Mohammed First, Oujda Morocco; 3 Department of Anesthesia-healthcare, Medical School, University Mohammed First, Oujda Morocco

**Keywords:** Kyste épidermoïde, Grande citerne, Quatrième ventricule, IRM de diffusion, Chirurgie, Epidermoid cyst, cisterna magna, fourth ventricle, Diffusion MRI, surgery

## Abstract

Les kystes épidermoïdes (KE) sont des tumeurs bénignes, congénitales, qui se développent à partir des inclusions ectodermiques. Les localisations dans la grande citerne et le quatrième ventricule sont très exceptionnelles. Nous rapportons, ici, le cas d'une patiente de 64 ans, admise pour des troubles de la marche, avec céphalées dont l'examen neurologique trouve un syndrome cérébelleux statique et un syndrome d'hypertension intracrânienne. Le diagnostic de kyste épidermoïde du quatrième ventricule et de la grande citerne fut évoqué sur les données de l'IRM en séquence diffusion puis confirmé par l’étude anatomo-pathologique. La patiente a bénéficié d'un abord direct avec une exérèse chirurgicale subtotale. Les suites opératoires ont été simples avec régression de sa symptomatologie clinique. Un suivi radio-clinique sur douze mois, trouve une patiente asymptomatique sans aucun signe de ré-évolution tumorale.

## Introduction

Les kystes épidermoïdes (KE), encore appelés « cholestéatomes primitifs » ou « tumeurs perlées de Cruveilhier », sont des tumeurs bénignes, congénitales, rares, développées à partir des inclusions ectodermiques [1]. Les localisations classiques sont: l'angle pontocérébelleux (la moitié des cas), la fosse temporale, la région supra-sellaire et la région quadrijumelle. Le siège au niveau de la grande citerne et celles dans le quatrième ventricule sont rares [2].

L'imagerie par résonance magnétique (IRM), en séquence de diffusion et en séquence écho gradient CISS 3D, constitue l'examen clé pour confirmer le diagnostic. Le problème de diagnostic différentiel se pose essentiellement avec les kystes arachnoïdiens. Le seul traitement est chirurgical.

## Patient et observation

**Figure 1:**
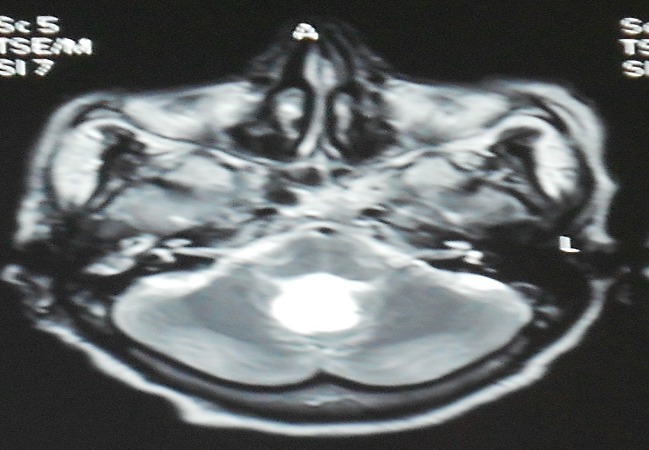
IRM cérébral en coupe axial séquence T2; montrant une lésion kystique hyperintense, au niveau de la grande citerne et le quatrième ventricule

**Figure 2:**
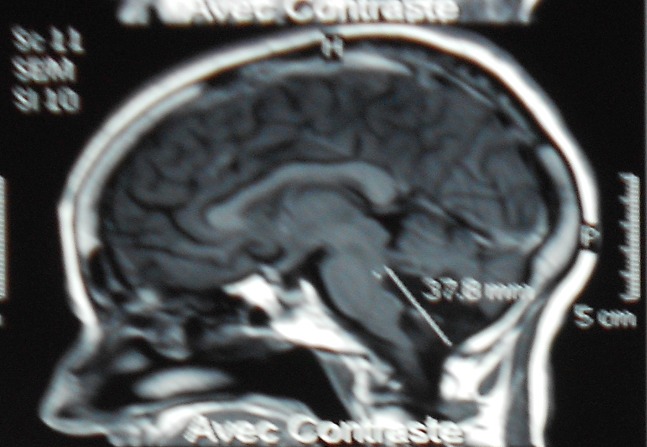
IRM cérébral en coupe sagittal séquence T1 gado; montrant une lésion kystique hypointense, ne prenant pas le contraste au niveau de la grande citerne et le quatrième ventricule

**Figure 3:**
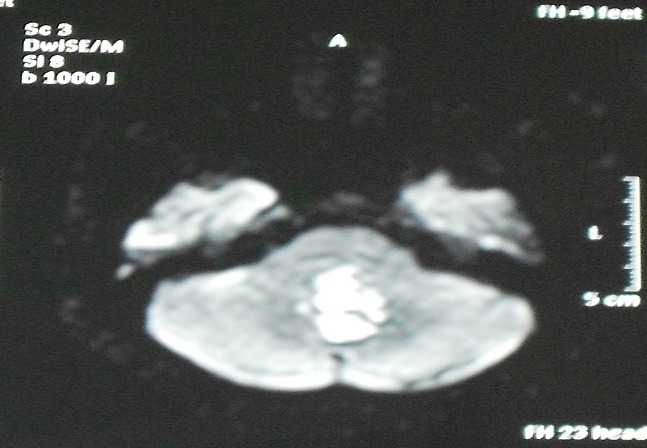
IRM cérébral en coupe axial, séquence de diffusion; montrant une lésion hyperintense, confirmant son caractère solide au niveau de la grande citerne et le quatrième ventricule

Une patiente âgée de 64 ans présentait, depuis 1 an et demi, des vertiges transitoires. Huit mois auparavant, le tableau clinique s’était aggravé par des troubles de l’équilibre avec une instabilité à la marche et des céphalées. L'examen à l'admission, trouvait une patiente consciente, sans déficit sensitivo-moteur, avec un discret syndrome cérébelleux statique. La TDM cérébrale objectivait une lésion se développant au niveau de la fosse cérébrale postérieur (FCP), médiane hypodense, ne prenant pas le contraste, avec une discrète hydrocéphalie. L'IRM montrait la présence d'une lésion médiane intra ventriculaire et au niveau de la grande citerne, de contours irréguliers, refoulant en avant la moelle allongée. Elle était en hypersignal en T2 (Figure 1), en hyposignal en T1 (Figure 2), et sans rehaussement après injection de produit de contraste. En imagerie de diffusion (Figure 3), elle était franchement hyper-intense. La patiente a été opérée en décubitus ventral, par un abord sous-occipital médian. Après une craniectomie sous-occipitale et l'ouverture de l'arc postérieur de C1, une exérèse subtotale de la lésion a été effectuée, avec persistance de la partie haute de la capsule. L'aspect peropératoire d'une « tumeur perlée », et l'examen anatomo-pathologique a conclu à un kyste épidermoïde. L’évolution a été marquée par une totale disparition des signes cliniques, un suivi radio-clinique a été préconisé. (Évolution sur 12 mois).

## Discussion

Les kystes épidermoïdes, encore appelés cholestéatomes primitifs ou « tumeurs perlées de Cruveilhier », sont des tumeurs bénignes, dysembryoplasiques, rares; ils constituent environ 1% de l'ensemble des tumeurs intracrâniennes primitives [1]. Développées à partir des inclusions ectodermiques [1]. Ils résultent le plus souvent d'un clivage incomplet de l'ectoderme neural et de l'ectoderme cutané, au moment de la fermeture du tube neural entre la troisième et la cinquième semaine de gestation, avec rétention de cellules ectodermiques dans le système nerveux [1, 3]. Ils peuvent, plus rarement, être secondaires à une pénétration post-traumatique ou iatrogène [4] de l’épiderme au niveau des espaces sous arachnoïdiens.

La fréquente latéralité des kystes épidermoïdes serait liée au développement concomitant des vésicules otiques et optiques [2]. Les localisations classiques sont l'angle pontocérébelleux (50%), la région sellaire et la fosse temporale [1], et ceux développés dans le V4 représentent 16% des cas; le siège au niveau de la grande citerne reste plus rare.

Malgré sa genèse au cours de la vie intra-utérine la découverte du kyste épidermoïde est tardive entre la 3ème et la 5ème décennie [1], comme c'est le cas de notre patiente. Sur le plan clinique, le syndrome cérébelleux est le maître symptôme, alors que le syndrome d'hypertension intracrânienne est moins fréquent, étant donné que l'hydrocéphalie est d'apparition tardive et ne se voit que dans moins de 50% des cas [1].

La probable persistance d'espaces d’écoulement du LCR entre la capsule et les parois du ventricule explique l'absence de corrélation entre l'importance du volume tumoral et la présence d'hydrocéphalie au moment de découverte de la tumeur [4].

Ces lésions sont faites d'une masse blanche nacrée, molle et entourée d'une capsule souvent adhérente aux parois adjacentes. Le contenu kystique est avasculaire et présente, à la coupe, un contenu jaunâtre, de consistance plus ou moins visqueuse, rappelant la cire de bougie et disposé en lamelles concentriques [5]. La lésion s'accroît lentement, et présente un caractère souple et déformable, s'adaptant aux espaces dans lesquelles elle évolue [6]. Les kystes s'accroissent par desquamation progressive des cellules épithéliales qui se transforment en kératine et en cristaux de cholestérine.

L'IRM en séquences conventionnelles (T1 et T2) montre classiquement une masse qui est respectivement de densité et de signal proches de ceux du liquide cérébrospinal sans ‘dème périlésionnel ni de prise de contraste, ainsi l'IRM en séquence flair, en imagerie de diffusion et en séquence echo gradient CISS 3D, permet une caractérisation aisée de la lésion [3].

En imagerie de diffusion, il existe une augmentation marquée du signal au sein des KE, cela traduit le caractère solide de la masse. Les problèmes de diagnostic différentiel avec les kystes arachnoïdiens et les kystes tumoraux sont contournés grâce à l'aspect hétérogène en séquence Flair, et surtout à l'aspect hyper-intense et hétérogène en séquence CISS-3D, mais néanmoins moins intense que celui du LCS. Par ailleurs, les contours des KE apparaissent irréguliers [3, 1].

L’évolution postopératoire des KE est habituellement simple; toutefois, une méningite aseptique peut survenir et engendrer une hydrocéphalie communicante, dont la prévention passe par l'exérèse totale tant que possible, l’éviction de la dispersion du contenu du kyste en peropératoire, ainsi que l'irrigation du foyer opératoire par de l'hydrocortisone [1]. Elle n'apparaît pas uniquement quand l'exérèse est incomplète, comme ce qui a été décrit dans la littérature [5], mais le seul passage, en cours d'intervention, du contenu du KE dans le LCS peut expliquer la survenue de la méningite chimique, même si l'exérèse totale comprend la capsule [6]. Elle peut être traitée par des ponctions lombaires répétées et une corticothérapie [7]. L'hydrocéphalie est habituellement spontanément régressive, nécessitant rarement la mise en place d'une dérivation.

Dans le cadre de la surveillance postopératoire, l'IRM en imagerie de diffusion permet une vérification précise du caractère complet ou non de l'exérèse [8]. Deux complications peuvent modifier l’évolution des KE: la rupture et la dégénérescence maligne [9]. La rupture kystique est la complication la plus fréquente, elle est le plus souvent secondaire aux manipulations chirurgicales, plus rarement spontanées et se manifeste par une méningite chimique aseptique. Quant aux formes malignes, elles sont rares et existent sous la forme de carcinomes malpighiens [10].

En cas d'exérèse incomplète, la croissance du résidu est aussi lente que celle de la tumeur native, elle nécessite néanmoins une surveillance annuelle, permettant d’évaluer son potentiel évolutif [1].

## Conclusion

Les kystes épidermoïdes du quatrième ventricule et de la grande citerne sont des tumeurs rares. En générale, ils atteignent une taille considérable avant de produire des symptômes. L'IRM en séquence de diffusion et CISS 3D confirme le diagnostique. Une exérèse la plus totale possible est recommandée. Cependant, en raison de leur nature bénigne et de leur évolution lente, une ablation incomplète de la capsule, au cas où elle serait adhérente au plancher du quatrième ventricule, peut être nécessaire afin d’éviter une morbi-mortalité lourde de conséquence. La chirurgie reste la seule mesure thérapeutique disponible.
